# Expanding pancreas donor pool by evaluation of unallocated organs after brain death: Study protocol clinical trial (SPIRIT Compliant)

**DOI:** 10.1097/MD.0000000000019335

**Published:** 2020-03-06

**Authors:** Yakup Kulu, Elias Khajeh, Omid Ghamarnejad, Mohammadsadegh Nikdad, Mohammadsadegh Sabagh, Sadeq Ali-Hasan-Al-Saegh, Silvio Nadalin, Markus Quante, Przemyslaw Pisarski, Bernd Jänigen, Christoph Reißfelder, Markus Mieth, Christian Morath, Benjamin Goeppert, Peter Schirmacher, Oliver Strobel, Thilo Hackert, Martin Zeier, Rainer Springel, Christina Schleicher, Markus W. Büchler, Arianeb Mehrabi

**Affiliations:** a Department of General, Visceral, and Transplantation Surgery, University of Heidelberg, Heidelberg; b Department of General, Visceral, and Transplant Surgery, University Hospital Tuebingen, Tuebingen; c Transplantation Center, Department of General and Visceral Surgery, Medical Center, Faculty of Medicine, University of Freiburg, Freiburg; d Department of Surgery, Universitätsmedizin Mannheim, Medical Faculty Mannheim, Heidelberg University, Mannheim; e Department of Nephrology, Heidelberg University Hospital, Heidelberg; f Institute of Pathology, University of Heidelberg, Heidelberg; g German Organ Transplantation Foundation, Frankfurt, Germany.

**Keywords:** histopathology, organ shortage, pancreas transplantation, unallocated pancreas graft

## Abstract

**Background:**

Pancreas graft quality directly affects morbidity and mortality rates after pancreas transplantation (PTx). The criteria for pancreas graft allocation are restricted, which has decreased the number of available organs. Suitable pancreatic allografts are selected based on donor demographics, medical history, and the transplant surgeon's assessment of organ quality during procurement. Quality is assessed based on macroscopic appearance, which is biased by individual experience and personal skills. Therefore, we aim to assess the histopathological quality of unallocated pancreas organs to determine how many unallocated organs are potentially of suitable quality for PTx.

**Methods and analysis:**

This is a multicenter cross-sectional explorative study. The demographic data and medical history of donor and cause of rejection of the allocation of graft will be recorded. Organs of included donors will be explanted and macroscopic features such as weight, color, size, and stiffness will be recorded by 2 independent transplant surgeons. A tissue sample of the organ will be fixed for further microscopic assessments. Histopathologic assessments will be performed as soon as a biopsy can be obtained. We will evaluate up to 100 pancreata in this study.

**Result:**

This study will evaluate the histopathological quality of unallocated pancreas organs from brain-dead donors to determine how many of these unallocated organs were potentially suitable for transplantation based on a histopathologic evaluation of organ quality.

**Conclusion:**

The comprehensive findings of this study could help to increase the pancreas graft pool, overcome organ shortage, reduce the waiting time, and also increase the number of PTx in the future. Registration number: ClinicalTrials.gov: NCT04127266

## 1 Introduction

With advances in surgery and immunosuppressive therapy, pancreas transplantation (PTx) has become an accepted and standardized therapeutic surgery worldwide. Today, PTx is a promising treatment for type 1 diabetes mellitus^[1–3]^ and for patients undergoing total pancreatectomy because of benign disease.^[4–6]^ It has been demonstrated that PTx can provide a good glycemic control and insulin independence and improve diabetic lesions including retinopathy, nephropathy, neuropathy, and vasculopathy.^[7,8]^ Furthermore, due to improved immunosuppressive agents, prophylaxis against infections and thrombosis, and modifications in surgical approaches, outcomes after whole organ PTx has consistently improved over the past 20 years.^[9–13]^ The 1- and 5-year patient survival rates after PTx are approximately 95% and 85%, respectively, and the 1-, and 5-year graft survival rates are 90%, and 70%, respectively.^[10,13,14]^

Morbidity and mortality still occur after PTx.^[15]^ Morbidity and mortality rates after PTx are mainly related to pancreas graft quality.^[16]^ To decrease these, some restricted criteria for pancreas graft allocation have been defined.^[17]^ However, these allocation criteria have decreased the overall availability of pancreas organs. Consequently, despite an increase in organs from deceased donors^[18]^, organ utilization (20% of all potential donor pancreata are ultimately used for whole organ transplantation) and also PTx rates (10% overall decline) have decreased.^[2,19]^ In the US, only 13% of deceased donors provide a pancreas that is utilized for transplantation.^[20]^ Data from Eurotransplant indicate that only 27% of donor pancreata are transplanted, either as whole pancreas grafts or as islet grafts.^[21]^ In addition to the restricted pancreas allocation criteria, some allocated/offered organs are not accepted by transplant surgeons (which is based on individual experience and personal skills) after an organ quality assessment.

Longer waiting lists, increased waiting times, and donor shortages have increased the need for and number of extended donor criteria (EDC) organs that are accepted for transplantation. To date, the most important selection criteria to identify suitable pancreatic allografts are donor demographics, donor medical history (age, gender, cause of death, etc), and the transplant surgeon's own organ quality assessment based on macroscopic appearance. However, it is unclear, whether unallocated organs have a poor histopathologic quality for transplantation. To the best of our knowledge, no systematic histopathologic quality assessment of unallocated pancreas grafts has been performed, so far. In this study, for the first time, we aim to assess the histopathological quality of unallocated pancreas organs from brain-dead donors to determine the number of unallocated organs that were potentially suitable for transplantation.

## 2 Methods

### 2.1 Study settings

The EXPLORE study is a multi-center cross-sectional explorative study. In this study, we aim to assess up to 100 unallocated pancreas organs. All evaluations and analyses are taking place at the Division of Transplantation at the Department of General, Visceral, and Transplantation Surgery, and the Institute of Pathology of the University of Heidelberg. This study was initiated on 01 November 2019 and is expected to last for 3 years. The study protocol was registered at ClinicalTrials.gov (registration number: NCT04127266).

### 2.2 Course of the study

**Figure 1:**
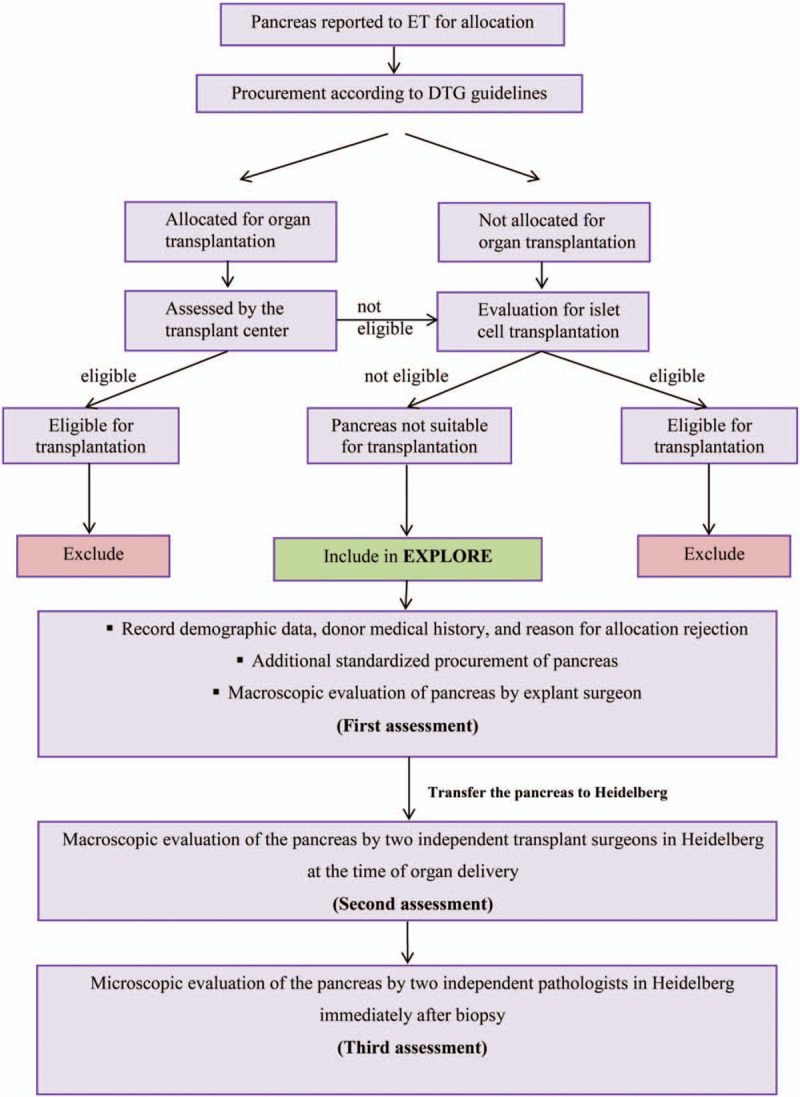
Study design flow chart. ET = Eurotransplant, DTG = German Transplantation Society.

As shown in the study flow chart (Fig. 1 and Table 1), all brain-dead pancreas donors in Baden-Württemberg, who reported to Eurotransplant for allocation, will be included in the study according to the organ donation regulations and laws in Germany and German Organ Transplantation Foundation (DSO) process instructions. Primary assessments will be performed by the certified procurement surgeons of 4 university hospitals in Baden-Württemberg (Heidelberg, Tübingen, Freiburg, and Mannheim) and pancreata will be explanted according to the procurement guidelines of the German Transplantation Society.^[22]^

**Table 1:**
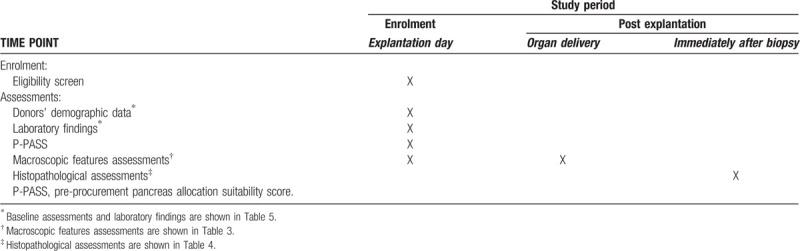
EXPLORE study design according to the SPIRIT checklist.

Organs which are allocated for transplantation will be transported to the accepting transplant center. If the pancreas is assessed suitable for solid organ transplantation by the transplant surgeon it will be excluded from the study. If the pancreas is not appropriate for organ transplantation, but consent to tissue donation is given and the pancreas is evaluated as suitable for islet cell transplantation by the responsible institution (Deutsche Gesellschaft für Gewebespende, DGFG), it will also be excluded from the study. Pancreata that are not allocated during the procurement operation and are also excluded for islet cell transplantation will be included in this study (Table 2).

**Table 2:**
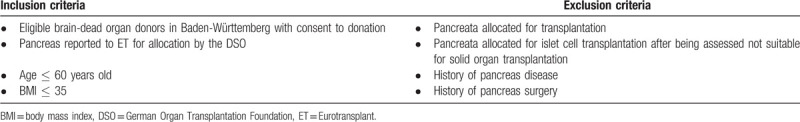
Inclusion and exclusion criteria of the EXPLORE study.

At time of organ procurement, macroscopic features such as weight, color, size, and stiffness will be recorded before and after cold perfusion by the macroscopic assessment form (first assessment, Table 3).

**Table 3:**
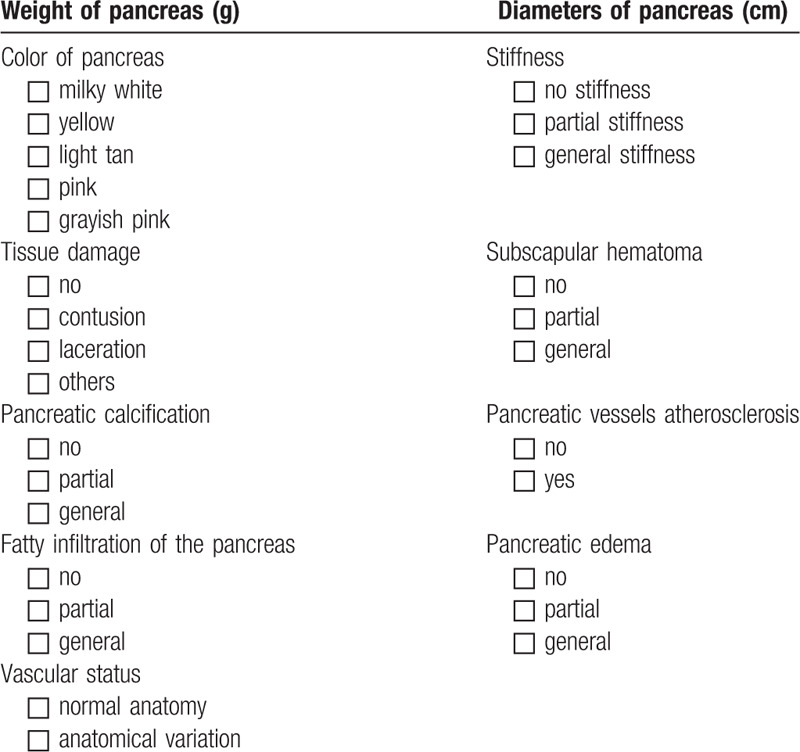
Macroscopic assessment of unallocated pancreas grafts (before and after cold perfusion) by transplant surgeons.

Afterwards organs will be explanted and sent directly to the Department of General, Visceral and transplantation Surgery of the University of Heidelberg. Then, the medical history of donors, whose pancreases are included in the study, will be evaluated. The donor medical history and reasons for organ rejection will be recorded. Biopsies will also be obtained from the head of the pancreas. Finally, a complete macroscopic assessment of the organs will be performed by two independent transplant surgeons (second assessment, Table 3) and microscopic evaluations will be done by two independent pathologists (third assessment, Table 4) at the Heidelberg University. A photo of the explanted pancreas (before and after cold perfusion) will also be made and saved for documentation.

**Table 4:**
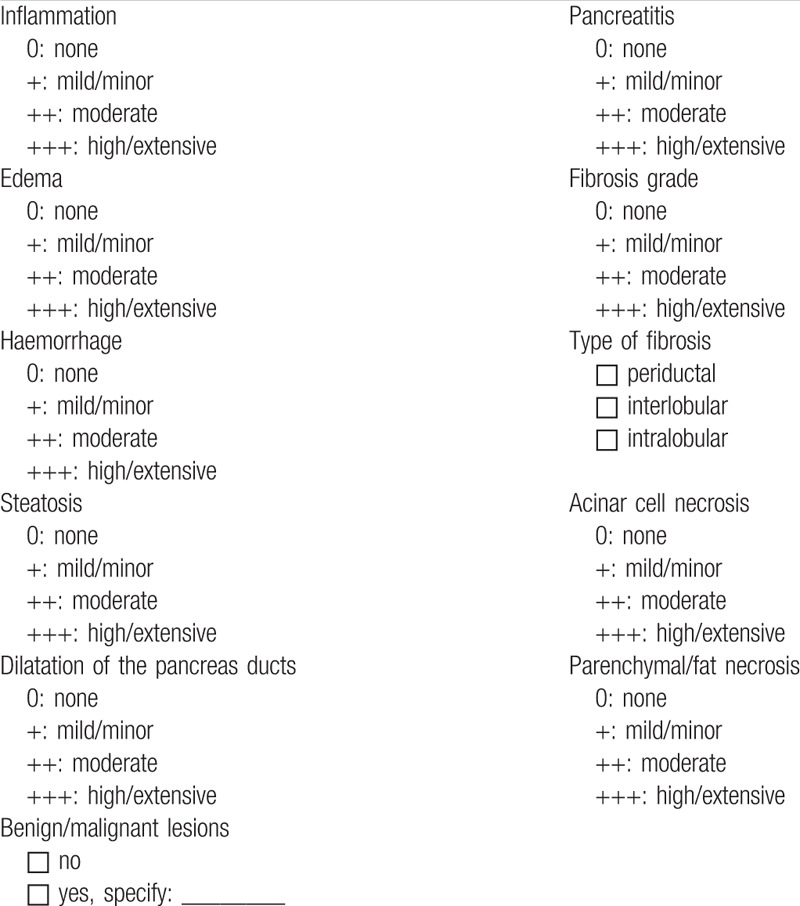
Histopathological assessment of unallocated pancreas organs.

### 2.3 Outcome measures

#### 2.3.1 Primary endpoint

In this multicenter study we will assess the histopathological quality of unallocated pancreas organs from brain-dead donors in Baden-Württemberg, Germany. Our aim is to determine how many of these unallocated organs were potentially suitable for transplantation based on a histopathologic evaluation of organ quality. We will evaluate the histopathologic features (third assessment) of unallocated organs, including the presence of pancreatitis, fibrosis, edema, hemorrhage, steatosis, dilation of pancreata ducts, and benign/malignant tumors, as soon as a biopsy can be obtained (Table 4).

#### 2.3.2 Secondary endpoints

Characteristics, medical history, and laboratory data of donors will be recorded. Additionally, macroscopic features of the pancreas organs, including weight, size, stiffness, color, and etc will be reported by the explant surgeon at the explant center and 2 transplant surgeons in Heidelberg (Table 3). Pre-procurement pancreas allocation suitability score (P-PASS)^[23]^ will also be calculated for each donor based on age, body mass index (BMI), intensive care unit (ICU) stay, duration of cardiac arrest/asystole, sodium, amylase, lipase, inotropic therapy [(nor)adrenaline or dobuta-/dopamine] (Table 5).

**Table 5:**
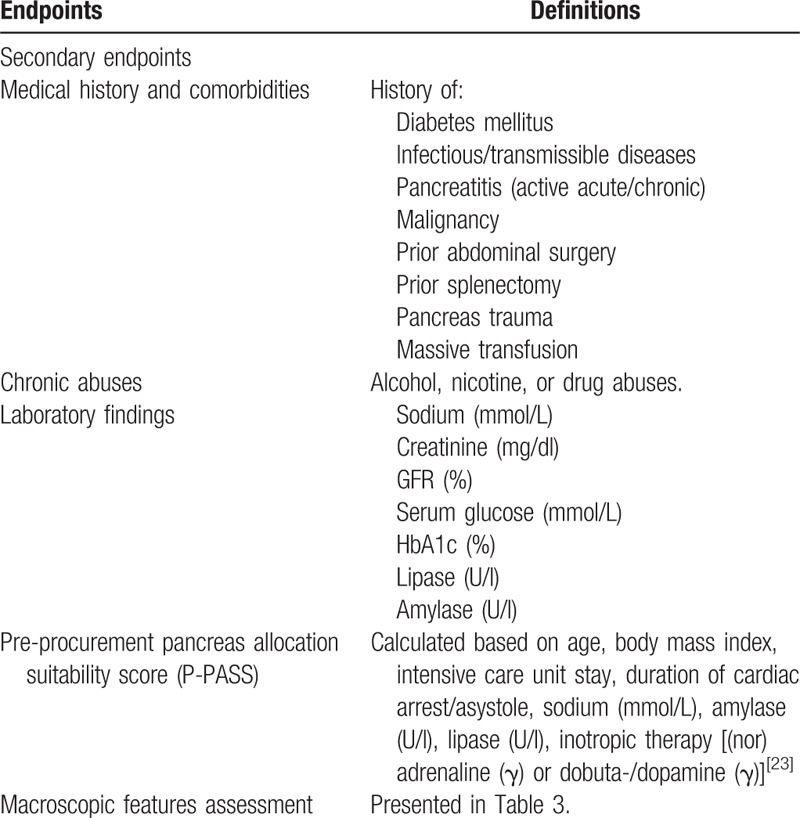
Secondary endpoints of the EXPLORE study.

### 2.4 Patient and public involvement

The patients and public were not involved in the planning of this study.

### 2.5 Modification of the protocol

Protocol amendments will be considered by the principal investigator. All protocol amendments will be submitted to the Ethics Committee for approval. No organ will be recruited until the modifications are accepted.

### 2.6 Methods for minimizing bias

To avoid biases macroscopic and microscopic evaluations of the organs will be performed by 2 independent transplant surgeons and 2 independent pathologists. Furthermore, selective reporting will be avoided by submitting the study protocol before data collection including all information concerning study endpoints and statistical analysis. Any financial relationship and any conflict of interest that may arise will also be declared.

### 2.7 Data management

All donor and graft data will be collected and recorded in case report forms (CRFs) by investigators before transfer to the data management center. To ensure accurate data collection, the CRF will be completed by an investigator who did not evaluate the donor and graft. All demographic and baseline clinical data, as well as primary and secondary outcome measures, will be recorded in the CRF. All CRFs will be given with an anonymous allocation number. The principal investigator will review and sign all completed CRFs.

### 2.8 Statistical design and analysis

#### 2.8.1 Sample size

This is an explorative cross-sectional study; therefore, we will not use a formal sample size calculation. We will evaluate up to 100 pancreata in this study.

#### 2.8.2 Statistical analysis

Continuous variables will be presented as means ± standard deviations. Categorical variables will be presented as percentages. Continuous variables will be analyzed between different reasons for allocation rejection groups using ANOVA or Kruskal–Wallis tests. Associations between categorical variables will be evaluated by chi-square or Fisher's exact test as appropriate. The significance level will be set at α ≤ 0.05, representing 95% confidence.

### 2.9 Ethics and dissemination

This protocol study received approval from the independent Ethics Committee of the University of Heidelberg (registration number: S-277/2019). Participants will voluntarily enroll in the study based on the consent to organ donation. In case of organs not being allocated the procedural instructions of the DSO recommend a pathological examination to validate the medical reasons given for organ refusal. In particular the histological examination of discarded organs could be relevant for other organ recipients of the same donor. The results of this study will be published in a peer-reviewed journal, and will also be presented at medical meetings.

## 3 Discussion

Restricted criteria for pancreas graft allocation are one of the major reasons for decrease in pancreas grafts, which are finally used for PTx. In addition to the restricted pancreas allocation criteria, a major part of allocated/offered organs are not accepted after assessments of the organ's quality by transplant surgeons. However, a qualitative study showed that assessment of medical donor characteristics is highly inconsistent when selecting an offered pancreas for transplantation.^[24]^ Loss et al^[25]^ analyzed the reasons for refusing organs in all Eurotransplant-registered German whole-pancreas donors between 2005 and 2009. Only 37% of offered pancreata were transplanted. However, interestingly, 62% of pancreata were of potentially high quality, and there were no clinically significant disparities between donors of used and unused pancreata, except age. However, organ quality was not validated using histopathological examinations by Loss et al. In this study, for the first time, we aim to assess the histopathological quality of unallocated pancreas organs, and to determine the proportion of these unallocated organs, which are suitable for PTx based on the histopathologic evaluations.

It seems that the most important selection criteria to identify suitable pancreatic allograft remain the donor/patient demographics and medical history (age, gender, cause of death, etc). To improve the post-PTx outcomes, some restricted criteria for pancreas graft allocation have been defined.^[17]^ But, several studies have suggested to use EDC organs for PTx.^[26,27]^ In an Eurotransplant dataset of 3666 deceased German donors (from 2002 to 2011), Drewitz et al^[28]^ showed that advanced age, high BMI, longer ICU stay, and the liver not being considered for procurement were the strongest predictors of pancreas non-transplantation. But, several transplant centers have reported good results with EDC that are older, have a higher BMI, or cardiac death.^[27,29–33]^ Proneth et al showed that selected organs of EDC donors aged >50 years can be used with outcomes similar to donors with standard-criteria organs.^[32]^ In this study, surgeons’ discretion regarding evaluation of the macroscopic organ quality appeared to be a major factor contributing to good outcomes when using older organs.

Comprehensive clinical assessment of the donors and histopathological assessment of the organs are the strengths of the present study. The outcome of present study can determine pancreata which can be used for organ selection for PTx and can lead to a significant expansion of the available pancreas donor pool and therefore decreased waiting time for PTx. However, in the EXPLORE study we do not assess the recipient outcome after PTx and therefore, further trials are needed to assess recipient outcome after using potentially suitable organs determined based on the present study. There is a limitation to the EXPLORE study which should be discussed. In this study we will evaluate the organs which are discarded to allocate according to the decision of the explant surgeon. However, a part of the pancreas organs are not allocated according to the second assessment of the transplant surgeon in the transplant center or due to the increased cold ischemic time, or pathological assessments in transplant center. Organs which are discarded in the transplant center, but not in the first assessment by explant surgeon, will not be included in the EXPLORE study.

In summary, despite contra opinions, the most commonly used selection criteria to identify suitable pancreatic allograft for PTx are based on demographic data, and medical history and discretion of the transplant surgeon is known as the major factor in selection of pancreas grafts. The EXPLORE study will be the first study which systematically evaluates the histopathological quality of unallocated pancreas organs. The comprehensive findings of this study could help to increase the pancreas graft pool, overcome organ shortage, reduce the waiting time, and also increase the number of PTx in the future.

## 4 Trials status

The EXPLORE study was initiated on 01 November 2019.

## Author contributions

**Conceptualization:** Arianeb Mehrabi, Yakup Kulu

**Methodology:** Arianeb Mehrabi, Yakup Kulu, Benjamin Goeppert, Peter Schirmacher, and Christina Schleicher

**Project administration:** Arianeb Mehrabi, Yakup Kulu

**Review and editing:** Silvio Nadalin, Markus Quante, Przemyslaw Pisarski, Bernd Jänigen, Christoph Reißfelder, Markus Mieth, Christian Morath, Benjamin Goeppert, Peter Schirmacher, Oliver Strobel, Thilo Hackert, Martin Zeier, Rainer Springel, Christina Schleicher, Markus W. Büchler, Arianeb Mehrabi

**Statistical design:** Elias Khajeh, Omid Ghamarnejad, Mohammadsadegh Nikdad, Mohammadsadegh Sabagh, Sadeq Ali-Hasan-Al-Saegh

**Writing – original draft:** Elias Khajeh, Omid Ghamarnejad, Mohammadsadegh Nikdad, Mohammadsadegh Sabagh, Sadeq Ali-Hasan-Al-Saegh

All authors read and approved the final manuscript.
